# Molecular Mechanism of Gibberellins in Mesocotyl Elongation Response to Deep-Sowing Stress in Sweet Maize

**DOI:** 10.3390/cimb45010015

**Published:** 2022-12-29

**Authors:** Bingying Leng, Ming Li, Chunhua Mu, Zhenwei Yan, Guoqi Yao, Xiangpei Kong, Changle Ma, Fajun Zhang, Xia Liu

**Affiliations:** 1 Maize Research Institute, Shandong Academy of Agricultural Sciences, Jinan 250100, China; 2 College of Life Sciences, Shandong Normal University, Jinan 250014, China; 3 College of Life Sciences, Shandong University, Jinan 250100, China

**Keywords:** sweet maize, deep-sowing, gibberellins, mesocotyls, GA20ox

## Abstract

Uneven germination is still a common problem in sweet maize planting. The mesocotyl is a key driver for ground-breaking sweet maize, and deep-sowing has a longer mesocotyl. However, the physiological and molecular mechanisms of sweet maize mesocotyl elongation in response to deep-sowing remain unknown. Here we found that sweet maize inbred line Ltx05 could obtain longer mesocotyls in deep soil of 10 cm depth, and that 20 mg/L GA_3_ was the optimal concentration to promote mesocotyl elongation and seedling emergence. Microstructure observation showed that the longitudinal cell length of mesocotyl at 10 cm sowing depth was significantly longer than that of 1 cm. Transcriptome analysis showed that microtubule process related differentially expressed genes may contribute to the longitudinal cell elongation. The content of GAs in the mesocotyl at 10 cm sowing depth was markedly higher than that of 1 cm. Combining transcriptome data and qRT-PCR at different developmental stages, *ZmGA20ox1*, *ZmGA20ox4* and *ZmGA20ox5* were identified as three positive regulation candidate genes during mesocotyl elongation under deep-sowing conditions, and this was further confirmed by the significant elongation of the hypocotyl in heterologous transformation of *Arabidopsis thaliana*. These results lay a foundation for improving the ability of sweet maize to tolerate deep-sowing stress and improving the breeding of excellent deep-sowing-tolerant germplasms.

## 1. Introduction

Because of its being high in nutrients such as proteins, vitamins, trace elements, cellulose, and folic acid and having the characteristics of grain, fruit, and vegetable, sweet maize has received extensive attention in recent years [1]. The energy provided by sweet maize is only 1/4 of that of kernel maize, and the sugar conversion rate is low; these make it widely loved by consumers. Due to insufficient accumulation of starch in the endosperm of sweet maize kernels, the seeds shrink after drying and lose water, becoming severely damaged. In addition, the high sugar content of the seeds and the large osmotic potential result in low seed vigor and emergence rates [2].

Due to the seed characteristics of sweet maize, seedlings of them are slender and weaker than those of common maize and are more suitable for shallow sowing. However, shallow-sowing cannot provide sufficient water to seeds, especially under drought and high-temperature conditions, a limitation which seriously affects sweet maize yield [3]. A deeper soil environment has more water than a shallow sowing environment [4] and can avoid chemical residues in shallow soil and damage caused to crops by wild animals [5,6], thereby stabilizing the primary form of the seed [7,8]. Further, due to the deeper distribution of crop roots, its drought resistance and lodging resistance can be enhanced [9]. Deep-sowing inhibits the root length and root total surface area of maize at the three-leaf stage, but the root growth rate of maize after the five- to seven-leaf stage under deep-sowing condition is faster than that of the shallow-sowing condition [10]. However, coupled with the germplasm characteristics of sweet maize itself, most of the existing sweet maize varieties have weak germination potential in the deep soil layer [11]. Therefore, improving deep-sowing tolerance is the key to increasing sweet maize yield.

Previous studies have shown that the mesocotyl is the key driver of seedling germination and establishment [2,6,9], and maize varieties with longer mesocotyls have increased topsoil germination rates and higher seedling emergence [12]. Furthermore, mesocotyl is the main area of maize to resist the invasion of soil-derived fungi and quickly respond to various abiotic stresses such as drought, low temperature, salt stress and waterlogging [13,14].

There are many factors that affect the elongation of mesocotyl, among which plant hormones are vital internal regulators [15]. Jasmonic acid (JA) may be involved in light-dependent regulation of mesocotyls [16]. JA inhibits mesocotyl elongation, and mutants with impaired JA synthesis pathways have a longer mesocotyl phenotype [17]. Ethylene inhibits gene expression in the JA biosynthesis pathway, reduces JA levels and promotes mesocotyl cell elongation [18]. Brassinolide (BR) attenuates the inhibitory effect of light on mesocotyl by activating *BZR1* to repress *GATA2* transcription and reduce the accumulation of GATA2 [19]. Indole acetic acid (IAA) mainly upregulates cell wall release activity, resulting in a continuous relaxation of the cell wall and mesocotyl elongation through cell growth [20]. It has been reported that the IAA-binding protein ABP1 is actively involved in the elongation of the deep-seeding maize mesocotyl [21].

Research on the regulation of mesocotyl elongation by gibberellins (GAs) has been increasing in recent years. Exogenous GA_3_ application promotes the elongation of wheat internodes, rice mesocotyl and germinal sheath, and Arabidopsis hypocotyl and roots [20]. GA regulates stalk elongation mainly by changing the orientation of cell microtubules, enhancing pectin methylation, and promoting cell elongation, which in turn promotes mesocotyl elongation [22,23]. In rice, exogenous GA_3_ can promote mesocotyl elongation, facilitate rapid seedling establishment and improve root early vigor, and GA combined with abscisic acid (ABA) can better promote mesocotyl elongation [24,25].

GA20ox and GA3ox are the main sites for plant growth and development and environmental signals to regulate GA biosynthesis pathway, while GA2ox mainly regulates GA biological activity, inactivating active GA, thereby regulating the content of GAs in plants [26,27]. The *ZmGA20ox1* gene overexpression line of maize showed increased biomass, plant height, stem length and longer leaf length under drought stress than WT [28,29]. The x rice *GA20ox2* mutant *sd1* showed significant decrease of plant height and GA content [30]. Overexpression of *AtGA20ox* gene in kenaf significantly increased the content of active GAs, cellulose content and fiber length and quality [31].

Previous studies mainly focus on the molecular mechanism of rice mesocotyl development. Thus, the molecular mechanism of maize mesocotyl elongation needs further exploration. GA_3_ treatment of maize seeds is a simple and feasible method to improve deep-sowing tolerance during germination [7]. In this study, to explore the role of GA in promoting mesocotyl elongation of deep-sowing sweet maize, we analyzed changes of phenotype and hormone content in sweet maize at different sowing depths. And we carried out the transcriptome data analysis and qRT-PCR data analysis. What’s more, *ZmGA20ox1*, *ZmGA20ox4* and *ZmGA20ox5* heterologous transformations to *A. thaliana* were also done.

## 2. Materials and Methods

### 2.1. Plant Materials and Culture Conditions

Seeds of sweet maize inbred line Ltx05 were used in this experiment and cultured in the greenhouse with a photoperiod of 16/8 h (day/night), a relative humidity of 65–70%, a light intensity of 600 μmol m^−2^ s^−1^ and a temperature of 28 ± 2/22 ± 2 °C (day/night).

### 2.2. Cultivation of Seedlings with Different Sowing Depths in Nutrient Soil

Seeds were cultured in nutrient soil at three sowing depths of 1, 5 and 10 cm with rectangular pots (51 × 37 × 14.5 cm, 30 seeds per pot) and watered every three days. The 15 days seedlings were used to determine the effect of different sowing depths on seedlings. Three replicates were performed for each group.

### 2.3. Treatment with Different Concentrations of GA_3_

The 1 cm and 10 cm sowing depths were chosen and separately treated with 0, 6, 10, 20 and 50 mg/L GA_3_ treatment solution for 7 days to determine the effect of GA_3_ on seedlings. Three replicates were performed for each group.

### 2.4. Treatment with GA_3_ and Gibberellin Inhibitor PP333

Seeds were surface-sterilized with 1% (*v*/*v*) sodium hypochlorite with constant shaking for 15 min and rinsed three times with distilled water before growth on sterilized filter paper in petri dishes (10 mm in diameter, 4 seeds per dish) for dark culture. The different groups were separately treated with 0, 20 and 50 mg/L GA_3_ and 200 mg/L PP333 (gibberellin inhibitor; polyconazole 15% wettable powder, WP), and the treatment solution was changed regularly and quantitatively every day for seven days. Ten replicates were performed for each group.

### 2.5. Measurement of Germination Rate, Mesocotyl Length and Root Surface Area

The germination rates of the different sowing depth groups were measured after 7 days. The mesocotyl and root systems of 15-day-old seedlings were rinsed with distilled water and dried with blotting paper. Mesocotyl length was measured with a ruler, and root morphology was imaged using a scanner (EPSON perfection V700 PHOTO, Dual Lens System, digital ICE technologies, Dhaka, Bangladesh).

### 2.6. Paraffin Sectioning Method

Paraffin sectioning was performed according to our previous study [32]. The mature zone of the mesocotyl parts was fixed with FAA fixative. Safranin and fast green were used to stain cut sections. After dehydration and mounting with neutral gum, the transect and slitting sections were observed using microscopic examination (Nikon Eclipse E100) and image acquisition analysis (Nikon DS-U3).

### 2.7. Determination of Endogenous Hormone Content

The endogenous hormone contents of 1 and 10 cm sowing depth mesocotyls were determined by LC-MS (Liquid chromatography–mass spectrometry) with three replicates performed for each group [33]. Quantitative samples were ground and added to an appropriate amount of internal standard (Olchemim/isoReag). Then, the powder was repeatedly extracted with acetonitrile solution in sequence. The supernatant was combined and fully reacted with TEA (triethylamine) and BPTAB (3-bromopropyl trimethylammonium bromide) and then blown dry with nitrogen. Finally, the samples were collected by filter membrane after re-solubilization with acetonitrile and later analyzed by LC-MS/MS. The MWDB (Metware Database) database was constructed based on the standards, and the data detected by mass spectrometry were analyzed qualitatively.

### 2.8. RNA Extraction and Transcriptomic Data Analyses

The total RNA from mesocotyl under 1 and 10 cm sowing depths at 15 days was extracted using TRIzol (Invitrogen, Waltham, MA, USA) and purified using the RNA easy Mini RNA kit (Qiagen, Hilden, Germany). High-throughput sequencing via an Illumina High-Seq2000 sequencing system was performed (Novogene Co., Ltd., Beijing, China), and genes with parameters of |log_2_(fold change)| > 1.5 and *p* < 0.05 were recognized as DEGs. Kyoto Encyclopedia of Genes and Genomes (KEGG) pathway analysis were also performed.

### 2.9. qRT-PCR Analysis of DEGs in Mesocotyls

qRT-PCR was carried out in a Light Cycler 480 (Roche) using SYBR Premix Ex Taq^TM^ (TaKaRa, Tokyo, Japan) according to the instructions. Mesocotyls at 7 and 15 days were used to measure the expression levels of candidate genes. The calculation of the relative expression value was performed according to the 2^−ΔΔCt^ method [34]. The qRT-PCR verification primers are shown in Table S1.

### 2.10. Plasmid Construction and Heterologous Transformation of ARABIDOPSIS Col-0

According to MaizeGDB (https://www.maizegdb.org/, accessed on 2 December 2022), the total length of coding sequence of *ZmGA20ox1*, *ZmGA20ox4* and *ZmGA20ox5* of inbred line Ltx05 was cloned and verified by sequencing (Supplementary Figure S1). The coding sequence of *ZmGA20ox1*, *ZmGA20ox4* and *ZmGA20ox5* without stop codon were inserted into pCAMBIA 3301 digested with Nco I. The constructs were introduced into the *Agrobacterium tumefaciens* strain GV3101 and then transformed into *A. thaliana* Col-0 by the Agrobacterium-mediated floral dip method [35]. After 0.1% herbicide (*v*/*v*) screening for three consecutive generations, homozygous Col 35S:: *ZmGA20ox1,* 35S:: *ZmGA20ox4* and 35S:: *ZmGA20ox5* were harvested and identified by qRT-PCR. Line OE1-3, OE4-11 and OE5-5 with lowest expression level were selected as the control (relative level is one) to calculate the relative expression level of different transgenic strains.

### 2.11. The Hypocotyl Observation and Determination in Transgenic Lines

Seeds of three *OE-ZmGA20ox1* (OE1-3, 1-10 and 1-9), *OE-ZmGA20ox4* (OE4-11, 4-9 and 4-8) and *OE-ZmGA20ox5* (OE5-5, 5-12 and 5-10) lines with low, medium, and high levels of *ZmGA20ox1, ZmGA20ox4* and *ZmGA20ox5* expression were placed on ½ MS medium (0.9 % agar) in square sterile culture boxes. Ten seeds per line were sown and ten biological replicates were performed. Homozygous seedlings cultivated for seven days were observed by dissecting microscope (Nikon, Tokyo, Japan) and hypocotyl length was measured.

### 2.12. Data Analysis

Experimental data were processed and analyzed with Duncan’s multiple range test or Student’s *t* test. All statistical analyses were carried out with SPSS v16.0.

## 3. Results

### 3.1. Sowing Depth Affects the Sweet Maize Emergence Rate and the Length of Mesocotyl

**Figure 1:**
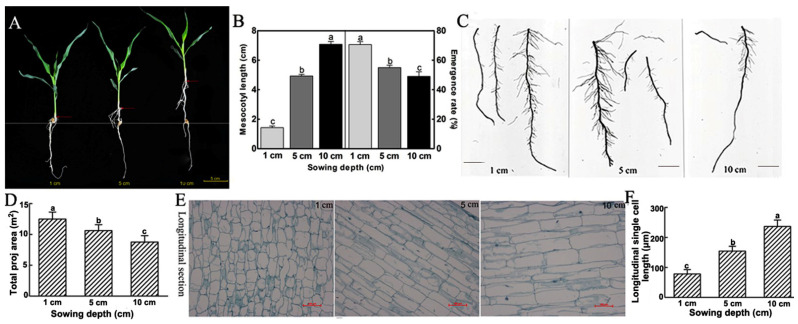
Differences of mesocotyl and root system of sweet maize inbred line Ltx05 under different sowing depth of 1, 5 and 10 cm cultivated for 15 days. The values are means ± SD of three replicates. Difference letters indicate significance using one-way ANOVA (*p* < 0.05). (**A**) The seedling phenotype and bar = 5 cm; (**B**) the mesocotyl length and emergence rate; (**C**) root system scanning and bar = 2 cm; (**D**) the root surface area; (**E**) longitudinal structure of mesocotyls; and (**F**) longitudinal single cell length.

The sowing depth of sweet maize was negatively correlated with the seedling emergence rate and positively correlated with the mesocotyl length (Figure 1A,B). In addition, with increasing sowing depth, the root surface area showed a decreasing trend (Figure 1C,D). These findings indicated that the growth of the root system was impacted by the sowing depth.

Deep-sowing is one of the main methods of breaking the soil, and sowing depth affects the length of mesocotyl longitudinal cells. Paraffin sections of the mature zone of the mesocotyl at the 15-day seedling stage at the different sowing depths of 1, 5 and 10 cm were made, and the results showed that with increasing sowing depth, the longitudinal cell sizes of the mesocotyls increased (Figure 1E,F). These findings reveal that the seedlings may achieve the purpose of breaking through the soil by increasing the longitudinal length of the mesocotyl cells.

### 3.2. Sowing Depth Affects GAs Content of the Sweet Maize Mesocotyl

**Figure 2:**
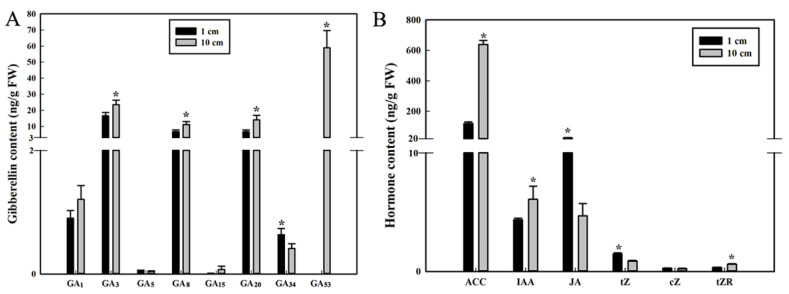
GAs contents (**A**) and other hormone (ethylene precursor, IAA, JA and cytokinin) contents (**B**) of Ltx05 mesocotyls under different sowing depth of 1 and 10 cm cultivated for 15 days. The values are means ± SD of three replicates. Asterisk indicates significant difference between 1 and 10 cm using a Student’s *t* test (* *p* < 0.05).

GAs have been reported to be involved in mesocotyl growth [7]. By measuring 16 types of GAs, eight endogenous GAs (GA_1_, GA_3_, GA_5_, GA_8_, GA_15_, GA_20,_ GA_34_ and GA_53_) were detected in sweet maize mesocotyl at the 1 cm versus the 10 cm sowing depth. Expect GA_1_, GA_5_ and GA_15_, the contents of GA_3_, GA_8_, GA_20_, and GA_53_ differed significantly between the two treatment groups. What’s more, except GA_34_, the contents of other GAs measured out in mesocotyl at the 10 cm sowing depth were all higher than these of 1 cm sowing depth (Figure 2A). These results suggest that GAs play a crucial role in the process of mesocotyl elongation in deep-sowing environments and GA_53_ may be a vital promoting factor in this process.

### 3.3. IAA, JA, Ethylene Precursor and Cytokinin Contents in the Mesocotyl in Relation to Sowing Depth

The content of cytokinin tZ in mesocotyl at the 1 cm sowing depth was higher than that of 10 cm sowing depth, and tZR showed the opposite trend. The content of IAA, ethylene precursor ACC in mesocotyl at the 1 cm sowing depth was significantly lower than that of 10 cm sowing depth and the JA content was lower in deep-sowing environments than in shallow sowing environments (Figure 2B). These results suggested that the IAA and ACC can promote the elongation of mesocotyls, while JA inhibits this process.

### 3.4. GA_3_ Promotes the Elongation of Mesocotyl and Seedling Emergence Rate in a Deep-Sowing Environment

**Figure 3:**
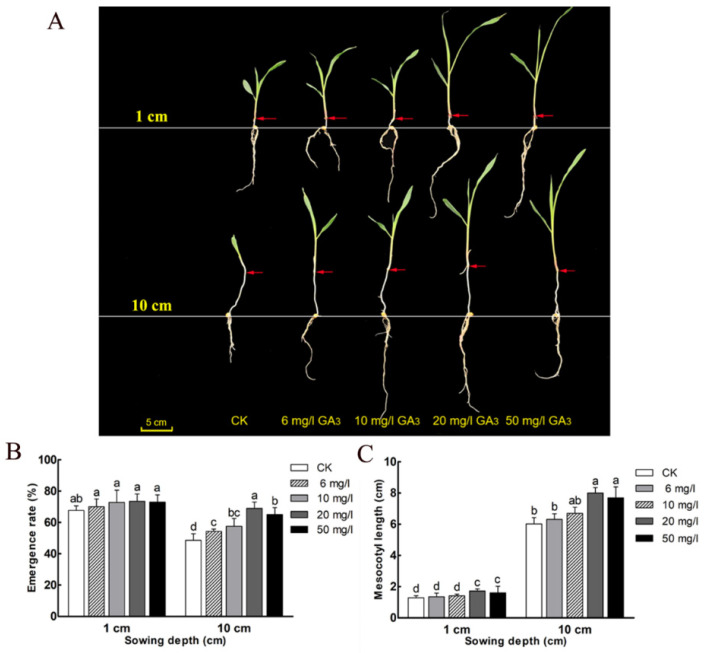
Morphological index (**A**), emergence rate (**B**), and mesocotyl length (**C**) of Ltx05 under different sowing depths (1 and 10 cm), treated with 0, 6, 10, 20 and 50 mg/L GA_3_ for 7 days. Data are means ± SD of three replicates, and different letters indicate significant difference at *p* < 0.05.

Seedlings from the 1 and 10 cm sowing depths treated with different concentrations of GA_3_ were investigated (Figure 3A). The results showed that under the 1 cm sowing depth, the emergence rate of the treatment groups with different GA_3_ concentrations had no difference. However, there were differences between the different GA_3_ treatment groups at the 10 cm sowing depth (Figure 3B). With increasing GA_3_ concentration (0, 6, 10, 20 mg/L), the emergence rate showed an increase trend. However, under 50 mg/L GA_3_ treatment, the emergence rate decreased significantly compared with the 20 mg/L treatment group. Mesocotyl length showed different trends (Figure 3C). The 6 and 10 mg/L GA_3_ treatment had no effect on mesocotyl elongation in both 1 cm and 10 cm sowing depth, whereas the 20 and 50 mg/L treatment increased the mesocotyl elongation (Figure 3C). Furthermore, the mesocotyl lengths of the different groups at the 10 cm sowing depth were significantly higher than the lengths of each group at the 1 cm sowing depth. These results suggested that 20 mg/L GA_3_ treatment can significantly alleviate the problem of seedling emergence rate reduction caused by deep-sowing.

### 3.5. GA_3_ Inhibitor Treatment under Dark Conditions Significantly Inhibits Mesocotyl Elongation and Germination Rate

**Figure 4:**
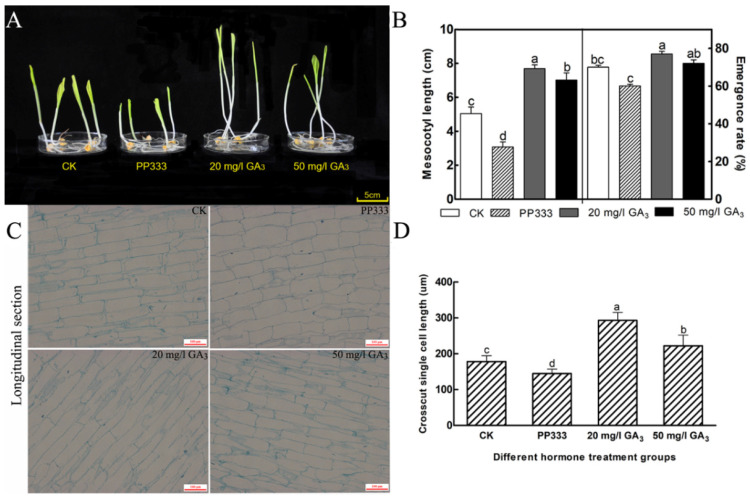
Differences of mesocotyl and emergence rate of Ltx05 under dark conditions treated with 0 mg/L GA_3_, PP333, 20 mg/L GA_3_ and 50 mg/L GA_3_ for 7 days. The values are means ± SD of ten replicates. Different letters indicate significance using one-way ANOVA (*p* < 0.05). (**A**) Morphological index; (**B**) the mesocotyl length and emergence rate; (**C**) longitudinal structure of mesocotyls; and (**D**) longitudinal single cell length.

Polycarbazole (PAC) enhances DELLA expression and inhibits GA biosynthesis and signaling pathway gene expression in maize under stress [36]. GA_3_ and GA_3_ inhibitors treatment in dark conditions were performed (Figure 4A). The results showed that PP333 (15% polyconazole WP) markedly inhibited seedling growth, while GA_3_ (20 mg/L, 50 mg/L) treatment promoted seedling growth. PP333 distinctly inhibited the elongation of the mesocotyl and the germination rate of seeds, while GA_3_ promoted them (Figure 4B).

Paraffin sections of mesocotyls of four dark-treated groups were generated separately. The longitudinal sections showed that GA_3_ played a vital role in promoting the cell elongation of mesocotyls, while PP333 played the opposite role. The cell longitudinal length of the mesocotyl of the 20 mg/L GA_3_ treatment group was obviously longer than that of the 50 mg/L GA_3_ group, followed by the 0 mg/L treatment group, and the shortest was observed in the PP333 treatment groups (Figure 4C,D). These data indicated that GAs played a critical role in the mesocotyl elongation of sweet maize.

### 3.6. Transcriptomic Analysis of Sweet Maize Mesocotyl under Different Sowing Depths

**Figure 5:**
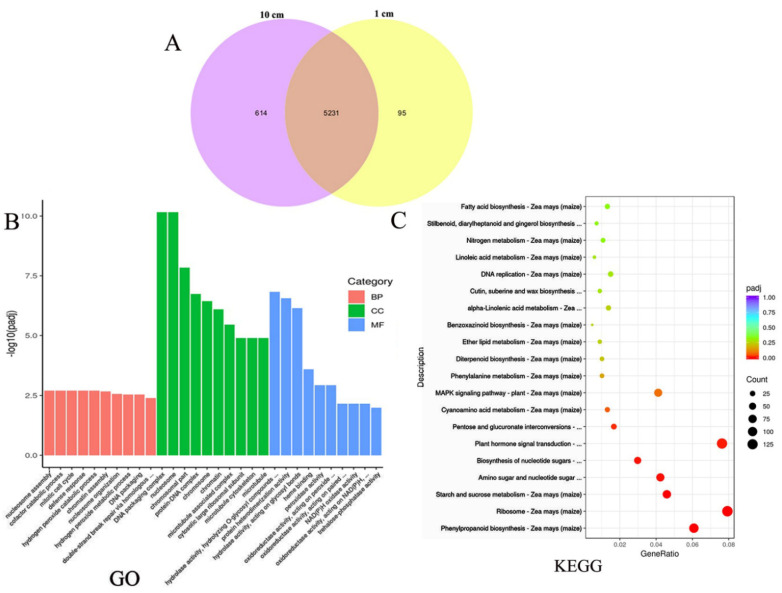
Analysis of transcription differences in mesocotyls of Ltx05 at 1 and 10 cm sowing depths. (**A**) DEGs identified under different sowing depths; (**B**) GO analysis; and (**C**) KEGG analysis.

Transcriptome analysis was performed on mesocotyls of sweet maize inbred line Ltx05 at 1 and 10 cm sowing depths. The results revealed that there were 5940 DEGs and a total of 5231 expressed under both 1 cm and 10 cm sowing depth, 95 were expressed under 1 cm sowing depth, and 614 were expressed under 10 cm sowing depth (Figure 5A).

GO analysis revealed that many DEGs between mesocotyls of different sowing depths were annotated to cellular components and many of them were associated with the cytoskeleton-related processes such as cytoskeleton, microtubule cytoskeleton and microtubule. Molecular function ontology showed that many DEGs were associated with processes such as hydrolase activity, peroxidase activity and oxidoreductase activity. Biological process ontology annotation showed that many DEGs were associated with hydrogen peroxide catabolic process, defense response and so on (Figure 5B). KEGG pathway enrichment analysis of the 1 and 10 cm mesocotyl DEGs revealed that 20 metabolic pathways were enriched (Figure 5C). Among them, DEGs mostly involved in phenylpropanoid biosynthesis, ribosome, starch and sucrose metabolism, amino sugar and nucleotide sugar metabolism, biosynthesis of nucleotide sugars and plant hormone signal transduction process. Plant hormone signal transduction process pathway enrichment showed that deep-sowing affected genes involved in the synthesis and metabolic processes of IAA, cytokinin, GA, ABA, ethylene, JA and so on (Supplementary Figure S2). These included a number of transcription factors and kinase, etc. such as EIL and AP2-EREBP transcription factors involved in ethylene, Aux/IAA and ARF transcription factors involved in IAA, GRAS and dwarf plant involved in GA, and many bZIP-transcription factors, bHLH-transcription factors and ZIM-transcription factors (Supplementary Figure S3). These results further illustrated that plant hormones play an important role in the mesocotyl response to deep-sowing stress.

### 3.7. GA-Related DEGs and qRT-PCR Analysis

**Figure 6:**
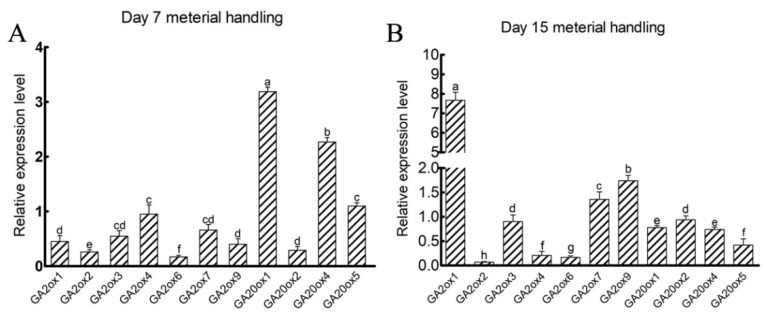
Relative expression levels determination of *GA2ox* and *GA20ox* genes in mesocotyls of Ltx05 at 1 and 10 cm sowing depths cultivated for 7 days (**A**) and 15 days (**B**) by qRT-PCR. The values are means ± SD of three replicates. Different letters indicate significant difference at *p* < 0.05.

*GA20ox-like* and *GA2ox-like* genes involved in GA synthesis and metabolism may be key genes regulating the growth of mesocotyl in sweet maize. Therefore, DEGs associated with GA were selected and seven *GA2ox*-like genes and four *GA20ox*-like genes were picked out for validation (Table S2). In addition to measuring the expression of GA related DEGs in mesocotyls of seedlings cultured for 15 days, the expression levels of GA related DEGs in mesocotyls of seedlings cultured for 7 days were also measured to explore the role of GA related DEGs in mesocotyl development. Among the 7 genes of *GA2ox* in mesocotyl at 15 days of growth, the expression trends of *GA2ox1*, *GA2ox2, GA2ox3*, *GA2ox4*, *GA2ox6* and *GA2ox7* were mainly consistent with the changes in transcription abundance except *GA2ox9* (Figure 6A). The expression trends of the four *GA20ox* genes (*GA20ox1*, *GA20ox2*, *GA20ox4* and *GA20ox5*) in the mesocotyl at the 10 cm sowing depth were lower than those of 1 cm sowing depth after 15 days of growth (Figure 6B). This was consistent with the changes in transcriptome identification.

Interestingly, the expression levels of the *GA2ox1*, *GA2ox7* and *GA2ox9* genes in the mesocotyl of seedlings cultured for 7 days at the 10 cm sowing depth were lower than those of control, which was contrary to the changes in seedlings cultured for 15 days. We hypothesized that the expression level of *GA2ox* (*GA2ox1*, *GA2ox7* and *GA2ox9*) was low in the rapid growth stage of mesocotyl, and increased after the end of mesocotyl elongation process, so as to reduce the content of active GA in plants. The expression of *ZmGA20ox1*, *ZmGA20ox4* and *ZmGA20ox5* showed higher levels in the mesocotyl of seedlings cultured for 7 days while decreased in mesocotyl of seedlings cultured for 15 days. These indicated that *GA20ox* genes (*ZmGA20ox1*, *ZmGA20ox4* and *ZmGA20ox5*) positively regulate mesocotyl elongation while *GA2ox* genes (*GA2ox1* and *GA2ox7*) showed the opposite role.

### 3.8. ZmGA20ox1, ZmGA20ox4 and ZmGA20ox5, in Varying Degrees, Promote the Elongation of Hypocotyls in Arabidopsis thaliana

Hypocotyl is an important part for *A. thaliana* seeds groundbreaking [37]. To verify whether *ZmGA20ox1*, *ZmGA20ox4* and *ZmGA20ox5* can promote plant groundbreaking ability, these three genes were overexpressed in Col-0. Transgenic lines with low, middle, and high expression of *ZmGA20ox1*: OE1-3, OE1-10, OE1-9; *ZmGA20ox4*: OE4-11, OE4-9, OE4-8; *ZmGA20ox5*: OE5-5, OE5-12, OE5-10 (Supplementary Figure S4) were selected to carry out further experiment.

**Figure 7:**
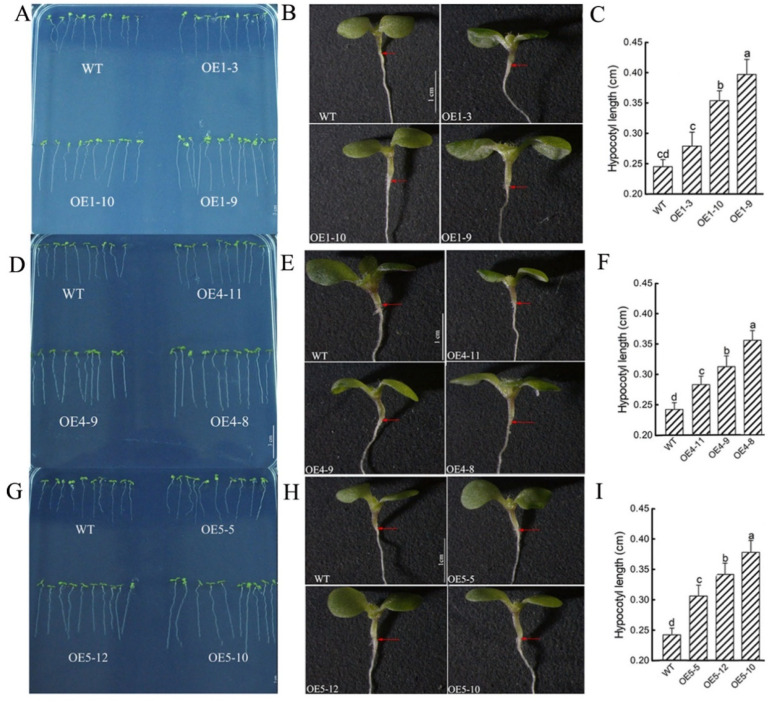
Hypocotyl development of Arabidopsis Col 35S:: *ZmGA20ox1*, 35S:: *ZmGA20ox4* and 35S:: *ZmGA20ox5*. Values of hypocotyl length are means ± SD of 20 seedlings. Different letters indicate significant differences at *p* < 0.05 according to Duncan’s multiple range test. (**A**–**C**) Phenotypes and hypocotyl length of WT and *OE-ZmGA20ox1* (OE1-3, 1-10 and 1-9); (**D**–**F**) phenotypes and hypocotyl length of WT and *OE-ZmGA20ox4* (OE4-11, 4-9 and 4-8); (**G**–**I**) phenotypes and hypocotyl length of WT and *OE-ZmGA20ox5* (OE5-5, 5-12 and 5-10).

The results showed that the plant growth of heterologous transformed lines was significantly better than that of WT. The hypocotyl length of OE1-9 and OE1-10 were markedly higher than that of WT while OE1-3 was a little higher than that of WT. Hypocotyl length increased with the increase of *ZmGA20ox1* expression level (Figure 7A–C). The hypocotyl length of OE4-8, OE4-9 and OE4-11 were higher than that of WT. And hypocotyl length also increased with the increase of *ZmGA20ox4* expression level (Figure 7D–F). The hypocotyl length of OE5-10, OE5-12 and OE5-5 were markedly higher than that of WT and was proportional to the expression level of *ZmGA20ox5* as well. (Figure 7G–I).

## 4. Discussion

As a popular maize species, more and more studies have been carried out on the planting and growth of sweet maize. Studies showed that seedling emergence is directly related to mesocotyl elongation [8,13]. Our study showed that with the increase of sowing depth, the emergence rate of sweet maize inbred line Ltx05 decreased and the mesocotyls elongated, which indicates that the seedlings sown deeply completed the germination by promoting mesocotyl elongation. The results of the mesocotyl paraffin section showed that in the 1 and 10 cm sowing depth environment, the sweet maize germination was mainly completed by the longitudinal elongation of the mesocotyl cells. The root system data show that the shallow sowing seedling may ensure the supply of water and nutrients by increasing the number of root hairs and expanding the contact area with the soil (Figure 1).

Phytohormones play an important role in mesocotyl elongation and growth of sweet maize [38]. Our determination of endogenous hormone content showed that deep-sowing can promote the synthesis of various plant hormones in the mesocotyl, especially IAA, GAs, ACC and tZ, so that the elongation of mesocotyl cells can be promoted. JA, instead, inhibited mesocotyl growth (Figure 2). These are similar to the results reported in maize and rice [16,36]. Studies showed that IAA treatment promoted cell elongation of mesocotyls under deep-sowing environments [20]. In rice seedlings, ethylene inhibits JA biosynthesis by decreasing the expression of *gaoyao1* gene and other genes to reduce JA levels and enhances mesocotyl elongation [18]. GA_3_ only or ethephon and GA_3_ treatment significantly promote the mesocotyl elongation of rice under 5 cm flooding depth stress [39]. Results in this study and these previous studies indicated that ethylene in sweet maize Ltx05 played a positive role in mesocotyl elongation under deep-sowing stress. The GA_53_ content was only detected in the mesocotyl at a sowing depth of 10 cm, which may be due to the soil environment influence and pressure during deep-sowing on the regulation of GA_53_-related signaling pathways.

Deep-sowing could reduce the emergence rate, but moderate GA_3_ treatment could alleviate the damage caused by this stress. GA_3_ had a better germination effect on seeds with low vigor. 20 mg/L GA_3_ was the best concentration to promote deep-sowing tolerance of sweet maize inbred line Ltx05 especially reflected in the improvement of germination rate (Figure 3). The inhibitor PP333 could significantly inhibit the promoting effect of GA_3_ on seedlings and mesocotyl elongation (Figure 4). The optimum concentration of GA_3_ for different maize varieties is also different [7,11].

Major life activities depend on differential gene expression [40]. Transcriptomic analysis revealed that sowing depth affected gene expression differences in mesocotyl. GO and KEGG analysis showed that deep-sowing stress affected the microtubule architecture, which ultimately affected the elongation of the mesocotyl and influenced the expression of oxidoreductase activity relative genes. Microtubules have been discovered to play an important role in mesocotyl elongation in rice [41]. Cortical microtubules destabilization induces mesocotyl cell elongation, and this process is related to endogenous GA content [42]. The hydrogen peroxide metabolic process also has been affected by sowing depth. A previous study showed that hydrogen peroxide pretreatment to seeds can improve deep-sowing stress tolerance of wheat [43]. KEGG enrichment showed that a large number of DEGs (especially transcription factors) were enriched in plant hormone signal transduction, and these were accordance with the difference of hormone content index measured in this experiment. In rice, the expression level of *OsTCP5* regulated by cytokinin and strigolactones (SL) is negatively correlated with the length of mesocotyl [44]. The expression of *ZmMYB59* decreases the germination and mesocotyl length of *Nicotiana tabacum* and rice [45]. The difference in mesocotyl phenotype and the change in hormone content at different sowing depths must be the result of the synergistic effect of these hormones on mesocotyl growth, and the specific regulatory pathways need to be further studied [13].

Physiological mechanism and genes involved in mesocotyl elongation have been extensively studied [46,47,48,49]. However, there are relatively few studies on the molecular mechanism of GA in the process of mesocotyl elongation under deep-sowing stress [34]. GA20ox played an important role in many life processes of the plant. The *GA20ox1* gene is highly expressed in wheat stems [50]. When *GA20ox* is overexpressed in rice, the plant exhibits internode elongation [51]. The *GA20ox1* gene is highly expressed in sorghum at the jointing stage [52]. Arabidopsis transgenic lines overexpressing *CsGA20ox1* evidently increase germination rate, rosettes area, leaf area and petiole length, and rosettes area and leaf area [47]. In our qRT-PCR results, the expression levels of the *GA20ox1*, *GA20ox4* and *GA20ox5* genes in the mesocotyl of 7 days seedlings at the 10 cm sowing depth were higher than those in the 1 cm control and lower than those in the 1 cm control of 7 days seedlings. The *GA20ox1*, *GA20ox4* and *GA20ox5* heterologous transformation of arabidopsis significantly increased the hypocotyl length and germination rate. These results suggested that *GA20ox1*, *GA20ox4* and *GA20ox5* may promote the growth of mesocotyl in the early stage.

## 5. Conclusions

**Figure 8:**
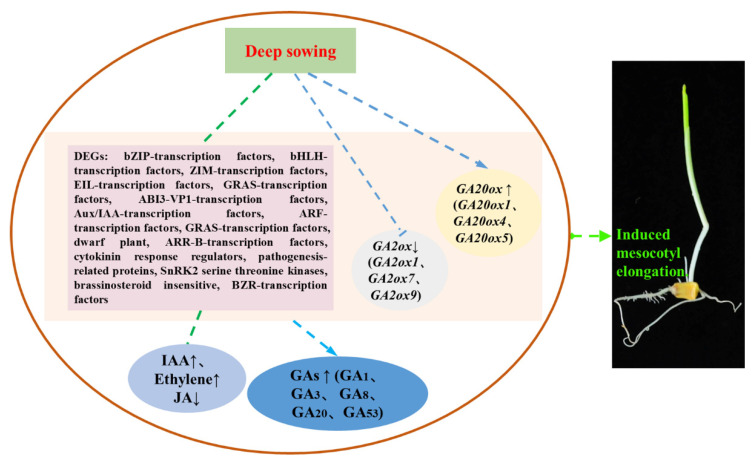
Molecular network of hormones in mesocotyl response to deep-sowing stress in sweet maize.

Based on the results of this experiment and previous studies, we summarized the role of hormones in mesocotyl, especially GA, in response to deep-sowing stress (Figure 8). Plant hormones played an important role in mesocotyl elongation under deep-sowing conditions, and GA played a positive regulatory effect. Deep-sowing stress significantly induced the differential expression of genes related to hormone signaling pathways, and genes associated with GA synthesis such as *ZmGA20ox1*, *ZmGA20ox4* and *ZmGA20ox5* act on mesocotyls in response to deep-sowing stress. *ZmGA20ox1*, *ZmGA20ox4* and *ZmGA20ox5* overexpression arabidopsis showed significant hypocotyl increase and this means overexpression of these three genes in sweet maize may also increase the mesocotyl length. These findings indicated that GA was of great significance for improving the tolerance of deep-sowing stress in sweet maize. Reasonable and appropriate GA_3_ treatment and to improve the expression of GA20ox genes by transgenic technology are of great significance for improving the poor and uneven seedlings of sweet maize under deep-sowing stress.

## Data Availability

Not applicable.

## Supplementary Materials

The following supporting information can be downloaded at: https://www.mdpi.com/article/10.3390/cimb45010015/s1. Figure S1: Nucleotide and amino acid sequences of ZmGA20ox1, ZmGA20ox4 and ZmGA20ox5. Figure S2: Hormone signal transduction process involved in hormone-related DEGs. Figure S3: Hormone-related DEGs in KEGG annotations. Figure S4: Expression levels of *ZmGA20ox1* (A), *ZmGA20ox4* (B) and *ZmGA20ox5* (C) examined using qRT-PCR in Col 35S:: *ZmGA20ox1*, 35S:: *ZmGA20ox4* and 35S:: *ZmGA20ox5*. Data are means ± SD of three replicates, and different letters indicate significant difference at *p* < 0.05; Table S1: Information of primers used in qRT-PCR. Table S2: Transcriptome analysis differences of DEGs associated with GA synthesis in 1 cm and 10 cm sowing depth at the 15-day seedling stage (10 cm/1 cm).
