# Supplementary material for: Molecular Mechanism of Gibberellins in Mesocotyl Elongation Response to Deep-Sowing Stress in Sweet Maize
Source: Curr Issues Mol Biol. 2022 Dec 29;45(1):197–211. doi: 10.3390/cimb45010015 (PMC9856927; doi:10.3390/cimb45010015)
Supplement: Supplementary file 1 [file cimb-45-00015-s001.zip › Figures S1¿CS4.pdf]

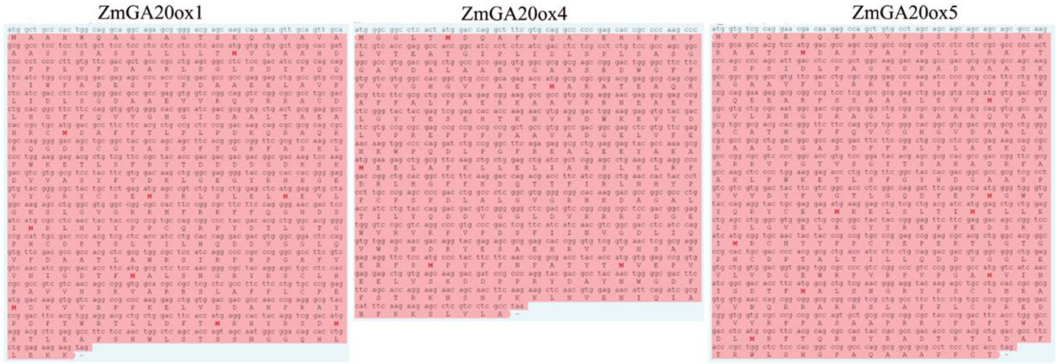

Figure S1 Expression levels of *ZmGA20ox1* (A), *ZmGA20ox4* (B) and *ZmGA20ox5* (C) examined using qRT-PCR in Col 35S:: *ZmGA20ox1*, 35S:: *ZmGA20ox4* and 35S:: *ZmGA20ox5*. Data are means ± SD of three replicates, and different letters indicate significant difference at P＜0.05.

Term	Description	Sample number	Background number	P-value	Corrected P-value	Gene_id	Gene_name	KEGG_ID
zma04075	Plant hormone signal transduction - Zea mays (maize)	128/1677	285/4750	0.000351608250205904	0.0070907663791524	Zm00001d007188 Zm00001d007395 Zm00001d026398 Zm00001d018326 Zm00001d012401 Zm00001d028974 Zm00001d022530 Zm00001d030310 Zm00001d028401 Zm00001d023242 Zm00001d011721 Zm00001d018178 Zm00001d002410 Zm00001d028936 Zm00001d027899 Zm00001d020614 Zm00001d019696 Zm00001d001945 Zm00001d018738 Zm00001d026594 Zm00001d047848 Zm00001d028793 Zm00001d033786 Zm00001d038698 Zm00001d033976 Zm00001d003601 Zm00001d033267 Zm00001d033339 Zm00001d038296 Zm00001d008201 Zm00001d022017 Zm00001d038326 Zm00001d043350 Zm00001d019364 Zm00001d047705 Zm00001d026540 Zm00001d010697 Zm00001d006753 Zm00001d043014 Zm00001d024783 Zm00001d043244 Zm00001d042886 Zm00001d005293 Zm00001d022542 Zm00001d002143 Zm00001d006677 Zm00001d011878 Zm00001d018734 Zm00001d013707 Zm00001d051889 Zm00001d013071 Zm00001d016294 Zm00001d030021 Zm00001d010082 Zm00001d000408 Zm00001d010411 Zm00001d020711 Zm00001d053642 Zm00001d018973 Zm00001d051302 Zm00001d031961 Zm00001d047563 Zm00001d015845 Zm00001d020606 Zm00001d012660 Zm00001d033319 Zm00001d044065 Zm00001d033050 Zm00001d039513 Zm00001d048046 Zm00001d043515 Zm00001d009296 Zm00001d005344 Zm00001d050365 Zm00001d053004 Zm00001d052543 Zm00001d016582 Zm00001d040536 Zm00001d009747 Zm00001d026690 Zm00001d012128 Zm00001d014297 Zm00001d048268 Zm00001d050018 Zm00001d038165 Zm00001d039163 Zm00001d022139 Zm00001d026653 Zm00001d003903 Zm00001d050768 Zm00001d005143 Zm00001d026530 Zm00001d026262 Zm00001d003659 Zm00001d043634 Zm00001d038436 Zm00001d043431 Zm00001d047053 Zm00001d034944 Zm00001d042463 Zm00001d016188 Zm00001d007448 Zm00001d033680 Zm00001d037774 Zm00001d042809 Zm00001d036215 Zm00001d014682 Zm00001d002533 Zm00001d025050 Zm00001d015778 Zm00001d049952 Zm00001d043117 Zm00001d002029 Zm00001d010658 Zm00001d031445 Zm00001d034463 Zm00001d018414 Zm00001d024160 Zm00001d004357 Zm00001d015521 Zm00001d046751 Zm00001d051911 Zm00001d048093 Zm00001d016277 Zm00001d009626 Zm00001d034920 Zm00001d026477 Zm00001d001865	EIL-transcription factor 2 - bZIP-transcription factor 113 - protein phosphatase homolog4 ethylene insensitive-like3 EIL-transcription factor 6 auxin import carrier1 - - brassinosteroid insensitive1a bZIP-transcription factor 4 - bZIP-transcription factor 52 ZIM-transcription factor 26 ZIM-transcription factor 28 cyclin10 ARF-transcription factor 4 pathogenesis related protein4 cytokinin response regulator2 - - - ARF-transcription factor 23 Aux IAA-transcription factor4 ABI3-VP1-transcription factor 17 bHLH-transcription factor 43 - bZIP-transcription factor 87 Aux IAA-transcription factor 34 - SnRK2 serine threonine protein kinase 11 - - - ARF-transcription factor 29 - - - bHLH-transcription factor 117 - protein phosphatase homolog3 cyclin11 bZIP-transcription factor 54 bZIP-transcription factor 27 BZR-transcription factor 7 - - Aux IAA-transcription factor 22 - Aux IAA-transcription factor 19 - - - - Aux IAA-transcription factor 38 - - Aux IAA-transcription factor 32 - histidine-containing phosphotransfer protein1 ethylene insensitive-like1 - - - Aux IAA-transcription factor 3 GRAS-transcription factor 46 ZIM-transcription factor 18 Aux IAA-transcription factor 7 ARR-B-transcription factor 1 Aux IAA-transcription factor 12 pathogenesis-related protein1 histidine-containing phosphotransfer protein2 ZIM-transcription factor 35 auxin import carrier2 bZIP-transcription factor 79 - bHLH-transcription factor 68 protein phosphatase homolog15 SnRK2 serine threonine kinase5 ARR-B-transcription factor 7 histidine kinase3 ZIM-transcription factor 14 bZIP-transcription factor 68 - protein phosphatase homolog14 ZIM-transcription factor 23 - ZIM-transcription factor 33 cytokinin response regulator7 - - - SnRK2 serine threonine protein kinase7 - - ARF-transcription factor 11 - - ARR-B-transcription factor 6 - - dwarf plant8 Aux IAA-transcription factor 28 auxin transporter-like1 MAP kinase7 - - ethylene receptor homolog2 - histidine-containing phosphotransfer protein3 bZIP-transcription factor 102 ZIM-transcription factor 32 bZIP-transcription factor 25 EIL-transcription factor 3 Aux IAA-transcription factor5 Aux IAA-transcription factor 24 bZIP-transcription factor 107 protein phosphatase homolog10 G2-like-transcription factor 24 bZIP-transcription factor 70 Aux IAA-transcription factor 18 - Aux IAA-transcription factor 23 protein phosphatase homolog9 AP2-EREBP-transcription factor 58 ZIM-transcription factor 34 cytokinin response regulator1	zma:103647946 zma:103648032 zma:100192007 zma:100281486 zma:103636504 zma:100285672 zma:103633580 zma:541998 zma:100284071 zma:541632 zma:103636232 zma:100284582 zma:100283318 zma:100274089 zma:100284778 zma:100384222 zma:100502214 zma:100383226 zma:542352 zma:541624 zma:103638597 zma:103634514 zma:732762 zma:100191784 zma:100284262 zma:103646418 zma:100281419 zma:100191280 zma:100284557 zma:103634643 zma:100285387 zma:100274512 zma:100383126 zma:103632200 zma:100281868 zma:103642166 zma:100280297 zma:100280445 zma:100383409 zma:103641361 zma:103651132 zma:100282657 zma:100285752 zma:100279481 zma:100502433 zma:100274111 zma:100272726 zma:103631918 zma:100274141 zma:103654108 zma:100286028 zma:100383950 zma:103637689 zma:100284059 zma:100279655 zma:100274569 zma:100193967 zma:100279520 zma:100280600 zma:103653849 zma:542586 zma:100279789 zma:103626859 zma:103632809 zma:100281196 zma:100216870 zma:103650192 zma:100284894 zma:100274580 zma:541639 zma:100283620 zma:100272820 zma:541625 zma:100285509 zma:103654551 zma:100383078 zma:103627198 zma:100382453 zma:100283932 zma:100193827 zma:103636390 zma:541642 zma:100284541 zma:100216617 zma:103630348 zma:103630796 zma:100276383 zma:100282637 zma:100274370 zma:541633 zma:109944035 zma:103642161 zma:100284202 zma:100282729 zma:103642983 zma:100216590 zma:100502387 zma:100282511 zma:100384298 zma:100285939 zma:100283610 zma:103649392 zma:100127531 zma:100191581 zma:100285200 zma:100286386 zma:103626407 zma:100273694 zma:541627 zma:100194025 zma:542596 zma:100501609 zma:100384736 zma:100274148 zma:100285594 zma:100381959 zma:100281448 zma:100192658 zma:103646787 zma:541638 zma:100279873 zma:100273242 zma:100283313 zma:100283352 zma:100381666 zma:100384381 zma:100383759 zma:541613

Figure S2 Hormone signal transduction process involved in hormone-related DEGs.

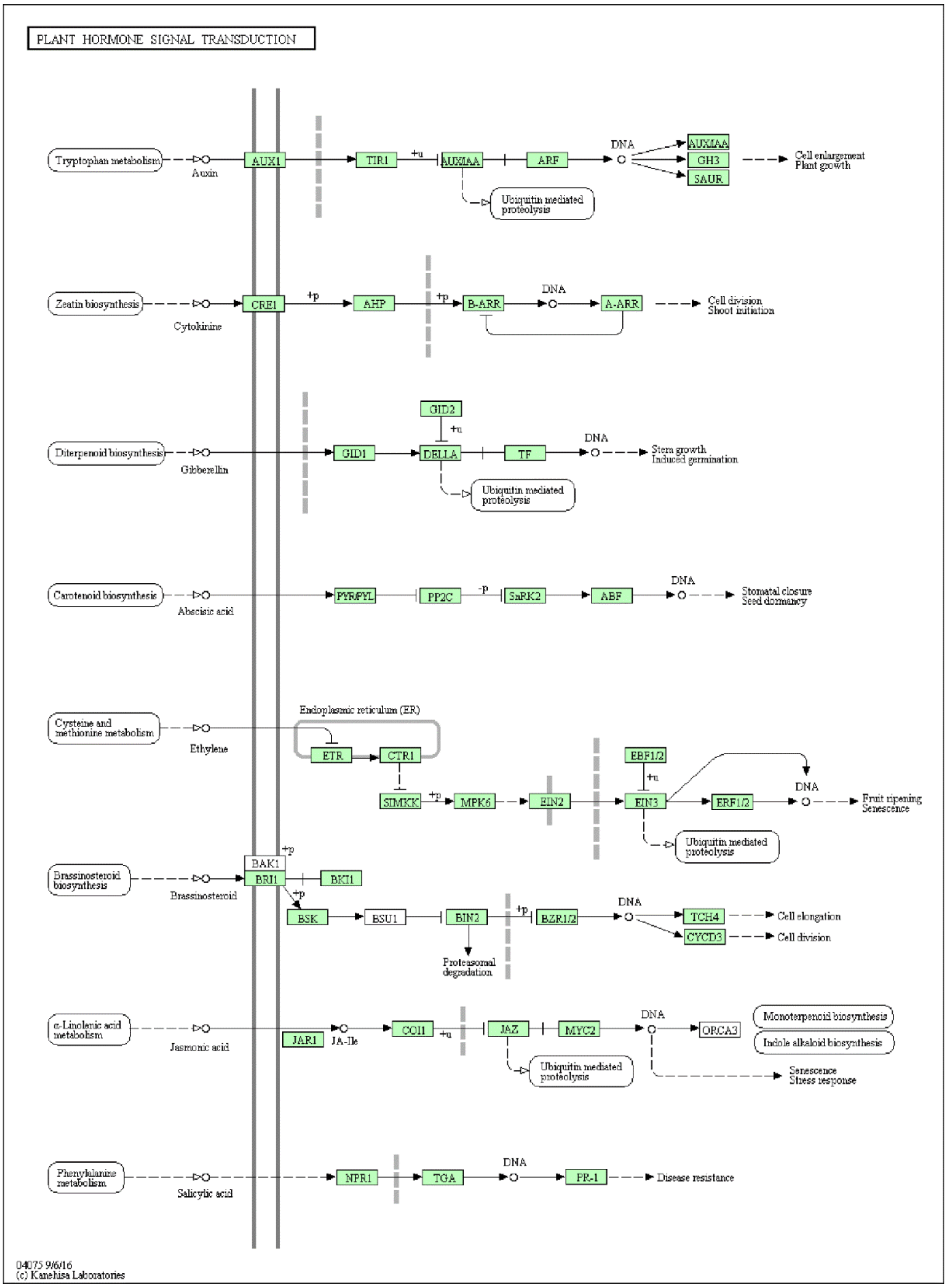

Figure S3 Hormone-related DEGs in KEGG annotations.

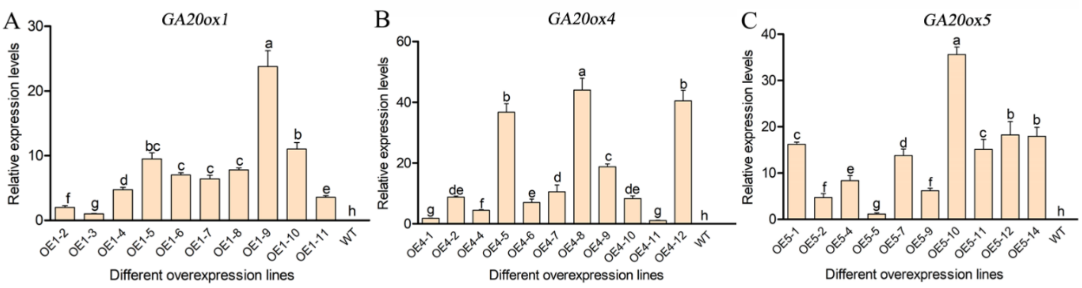

Figure S4 Nucleotide and amino acid sequences of ZmGA20ox1, ZmGA20ox4 and ZmGA20ox5.
