# Supplementary material for: Molecular Mechanism of Gibberellins in Mesocotyl Elongation Response to Deep-Sowing Stress in Sweet Maize
Source: Curr Issues Mol Biol. 2022 Dec 29;45(1):197–211. doi: 10.3390/cimb45010015 (PMC9856927; doi:10.3390/cimb45010015)
Supplement: Supplementary file 1 [file cimb-45-00015-s001.zip › Tables S1¿CS2.pdf]

Table S1 Information of primers used in qRT-PCR.

Genome ID	Gene name	Forward primer sequence	Reverse primer sequence
Zm00001d037565	*GA2ox*1	GAGTATGTGGGAGCGATGAG	GGTGGAGGGTAGTGGTTTATC
Zm00001d002999	*GA2ox*2	ACAACCTGTACAAGAGCGTG	CGAAGGTGAAGTCTCTGTACG
Zm00001d043411	*GA2ox*3	GAGGTTCAGGAGCGTGAAG	CCGCGAAGTAGATGAAGGAAAC
Zm00001d017294	*GA2ox*4	AACAGATACAAGAGCGTGGAG	GAAAGTAGGCGACGGAGTAG
Zm00001d037724	*GA2ox*6	AAACCACCCTCCCAATCATC	TCGCCGACATTGACGAAG
Zm00001d038695	*GA2ox*7	GGGTGTCCATGATCTACTTCG	GTAGTCGCCCCATGTGAAG
Zm00001d008909	*GA2ox*9	CTGCGGGTGAACCACTAC	GAGCACCGAGATGATCTGC
Zm00001d034898	*GA20ox*1	CTACTTCGTGGACAAGCTGG	ACAGACGGCTCATCTCAGAG
Zm00001d007894	*GA20ox*2	AAAATGCAGGGAGGTGTACC	TGGTTCAGCCGCATGAC
Zm00001d013725	*GA20ox4*	CTGGTGAGCAAGGACGATC	TTGAAATGCGCGATCTGAATG
Zm00001d012212	*GA20ox5*	CTCCCCTGTTACAAATACCCC	CTGGCTCTTGTCGTTCCTG

Table S2. Transcriptome analysis differences of DEGs associated with GA synthesis in 1 cm and 10 cm sowing depth at the 15-day seedling stage (10 cm/1 cm).

**Swissprot Description**	**Gene-id**	**log_2Fold Change**	**pvalue**	**padj**
ZmGA2ox1	Zm00001d037565	4.668910698	0.000706654	0.003521947
ZmGA2ox2	Zm00001d002999	-2.532823236	8.02E-28	1.30E-25
ZmGA2ox3	Zm00001d043411	-1.60336952	1.41E-07	1.78E-06
ZmGA2ox4	Zm00001d017294	-2.395064914	4.83E-16	2.36E-14
ZmGA2ox6	Zm00001d037724	-2.479127089	2.71E-31	5.49E-29
ZmGA2ox7	Zm00001d038695	3.784351874	0.000177668	0.001067743
ZmGA2ox9	Zm00001d008909	-4.733853057	8.24E-81	3.04E-77
ZmGA20ox1	Zm00001d034898	-1.724877624	1.42E-14	5.83E-13
ZmGA20ox2	Zm00001d007894	-2.49328791	0.004483223	0.016943248
ZmGA20ox4	*Zm00001d013725*	-2.110525696	0.000105074	0.000672358
ZmGA20ox5	Zm00001d012212	-1.909198573	1.89E-10	4.05E-09
